# Coaching Patients to Understand and Use Patient-Reported Outcome Data: Intervention Design and Evaluation

**DOI:** 10.2196/65931

**Published:** 2025-06-30

**Authors:** Martha Burla, Brocha Z Stern, Andrew BL Berry, Sarah Pila, Patricia D Franklin

**Affiliations:** 1 Department of Medical Social Sciences, Feinberg School of Medicine, Northwestern University, 625 N Michigan Ave, Chicago, IL, 60611, United States, 1 312-503-4348; 2 Departments of Population Health Science and Policy and Orthopaedics, Icahn School of Medicine at Mount Sinai, New York, NY, United States

**Keywords:** osteoarthritis, shared decision making, patient coaching, patient-reported outcomes, surgical decisions, health status, arthritis, arthritis care, shared knowledge, clinical consultation, quantitative evaluation, self-assessment, self-efficacy, patient empowerment, coaching intervention, health information, decision aid, patient support

## Abstract

**Background:**

Providing patients with information about their health and treatment options is important to ensure care that best reflects patient needs, values, and preferences. Patient-reported outcomes (PROs), measures of health status, are regularly collected in clinical contexts and scores can be returned to patients in personalized decision aids. One example of a PRO-based decision aid is the Arthritis care through Shared Knowledge (ASK) report, which shares individual PRO data on knee and hip arthritis–related pain and functional limitations with patients. However, given that the use of such data in clinical consultations is unfamiliar to many patients, support may be required to ensure this information is understood and used as intended.

**Objective:**

This paper describes ASK coaching, an online 1-hour group session designed to ensure patients understood the ASK report, including their PRO scores, and how to use the information in conversations with their clinicians. We present (1) quantitative evaluation results associated with attendance and self-assessment of learning and (2) qualitative evaluation results on motivation to attend, acceptability of the session format, and achievement of session goals.

**Methods:**

The session was designed and refined collaboratively with clinical experts and patient advisers. Patients in one arm of a pragmatic cluster-randomized trial evaluating the ASK report were invited to attend this session. To understand the profile of attendees (N=438) sociodemographic and clinical data were compared with all participants invited to coaching (N=1545) and a patient-reported assessment of self-efficacy was collected on a subset (N=692). In addition, a postsession survey was used to self-assess learning. Qualitative data were synthesized from semistructured postcoaching interviews, paired pre- and postcoaching interviews, and free-text responses to a postsession survey. A qualitative descriptive approach was used for analysis.

**Results:**

Compared with nonattendees, patients reporting higher education, greater health literacy, Medicare insurance, and lower self-efficacy for managing treatments were more likely to attend ASK coaching when invited. Participants’ self-assessment of learning showed an improved understanding of current and projected osteoarthritis symptoms and where to find additional information. Qualitatively, patients reported attending coaching to gain information that could benefit their treatment or aid in research. The online group format was generally described as acceptable, and the session goals related to understanding the report and preparing for future conversations with clinicians were met. Suggestions for improvement, such as providing more opportunities for within-group interaction, were also provided.

**Conclusions:**

Our results highlight the value of coaching as an intervention to help patients understand and use novel health information, including PRO data, in conversations with clinicians. Given that it was well-liked by patients, promoting a greater understanding of the PRO-based ASK report, and increased feelings of preparedness for clinical consultation, coaching appears to be a promising intervention to support patients in understanding and using their personal health data.

## Introduction

Patient-reported outcomes (PROs)—standardized assessments of patients’ pain, function, and health-related quality of life—are increasingly collected in routine clinical settings [1,2]. PRO scores reflect the patient’s perspective on their health and can be used to guide collaborative patient-clinician conversations and care decisions [3,4]. Specifically, PRO scores can be embedded into decision reports used with clinicians at the point of care or accessed via the electronic patient portal and used independently by patients to prepare for a clinical consultation [5,6]. Such uses of PRO data may be particularly important when managing a progressive chronic disease like osteoarthritis. Since patients’ gradual decline may lead to unrecognized impairments, PRO information can help them self-reflect on their current health and symptoms [3,7]. Furthermore, incorporating patient-reported data into a clinical encounter has been shown to improve communication between patients and clinicians [8], which is highly relevant for collaborative conversations and care decisions in chronic conditions including osteoarthritis.

The need to refine decision-making in total joint replacement, a common treatment for osteoarthritis, has been specifically acknowledged due to its status as a high-volume elective surgery with known disparities in usage [9-12]. In response, several PRO-based decision aids have been developed to support decision-making for hip and knee osteoarthritis [13-15]. One such report is the Arthritis care through Shared Knowledge (ASK) report, which shares personalized PRO data on current pain and function as well as estimated postoperative symptom information, with patients and clinicians [16]. Detailed information about the content and design of the report, as well as clinician and patient perspectives on report use, are available elsewhere [16-19].

While patients may want to use decision aids, like the ASK report, to engage in conversations about their care, they do not always feel able to do so. Instead, some patients report that they fear upsetting physicians who might perceive patient self-advocacy as a challenge to their authority [20]. In addition, the independent use of personal health data is new to many patients, and in the case of the ASK report specifically, they may experience difficulty understanding what PRO scores represent, and how they can be used in clinical conversations and care decisions [18,21]. Last, in patient interviews completed during the development of the ASK report, patients suggested that additional education may be helpful to ensure patients can independently use personal health data in clinical conversations and feel comfortable doing so.

“Coaching,” a form of nondirective support provided by a trained individual, has been discussed as part of supporting patients in combination with decision aids [22]. Coaching can occur outside of clinical consultations and may have utility in supporting patients to optimally understand and use personal health data in conversations with clinicians. As such, ASK coaching was created and embedded within a pragmatic trial of the ASK report to support patients in (1) understanding their personal data, including the PRO scores, and (2) using that data in conversations with clinicians. This paper describes the ASK coaching session design process and presents the results of a multi-method evaluation of the coaching intervention.

## Methods

### Overview of the Parent Study

**Figure 1.**
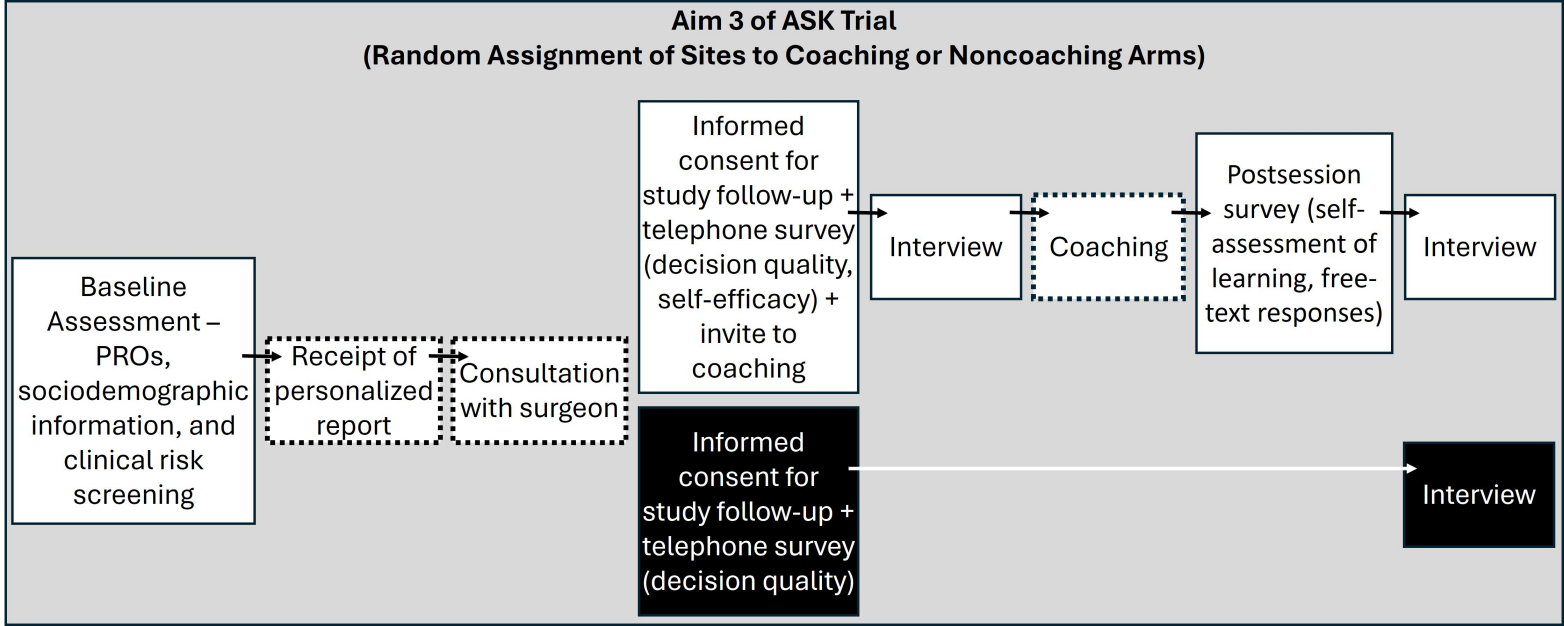
The figure outlines the primary components of the study procedures for aim 3 (May 2020-February 2022) of a pragmatic cluster-randomized trial for patients with hip and knee osteoarthritis. The noncoaching arm (black box) is not the focus of this paper. The boxes with dashed borders reflect study activities that do not involve data collection. ASK: Arthritis care through Shared Knowledge.

The ASK study was a pragmatic cluster-randomized trial with the primary aim of evaluating the effectiveness of a personalized PRO-based report containing estimates of likely outcomes of surgery if elected to treat advanced hip and knee osteoarthritis (ClinicalTrials.gov identifier: NCT03102580). A copy of the ASK report is in Multimedia Appendix 1. The study enrolled patients who scheduled an initial visit with one of 36 orthopedic surgeons to discuss knee and hip arthritis treatment options, including total joint replacement surgery. Aim 1 involved report co-design, with patients and clinicians, aim 2 aimed to compare decision quality for patients at sites randomized to receive or not receive the report, and aim 3 included adding a patient coaching intervention (“ASK coaching”) to a randomly assigned subset of study sites. Figure 1 depicts study procedures during aim 3. In this aim, patients completed PROs prior to their initial office-based consultation with a participating orthopedic surgeon. Within the month following this initial consultation, study staff contacted all patients via telephone to invite participation in the study and assess decision quality. Participants receiving care in study sites randomized to coaching were also invited to attend an ASK coaching session at this time.

### Coaching Intervention Co-Design and Iteration

During the design of the parent study, patient advisers who had recently made decisions regarding the treatment of their hip or knee osteoarthritis were consulted [16]. A subset of those patients expressed that an opportunity to review the content of the ASK report in a group setting would be helpful. The ASK coaching intervention was included in the study design based on this recommendation and, like the ASK report, was developed in collaboration with patient partners and clinical stakeholders.

The research team determined that the coaching intervention would focus on helping participants understand the personal data provided in the ASK report and how to use that data in future conversations with clinicians. The research team then consulted with Citizens for Patient Safety (CPS), an organization that educates patients and clinicians about medical decision-making and navigating the health care system. CPS recommended further helping patients prepare for clinical consultations by encouraging them to be active members of the health care team and helping them develop a list of questions to ask clinicians. They specifically highlighted the Agency for Healthcare Research and Quality’s (AHRQ) “Questions are the Answer” materials [23] as a potentially useful tool to guide the session.

A trained health educator (an individual with a Master of Public Health degree and a certification in health education) then developed the hour-long online group coaching session and an osteoarthritis-tailored patient handout based on the AHRQ materials recommended by CPS. This handout, entitled “I Have a Voice” (Multimedia Appendix 2), was used throughout to help patients reflect on their data and how it might inform future conversations with clinicians. The research team also created a website consolidating additional patient resources from reputable sources, including AHRQ, Arthritis Foundation, American Academy of Orthopaedic Surgeons, and American Association of Hip and Knee Surgeons. After completing this initial design, the research team met with clinical experts, including a patient advocacy leader from CPS, a senior orthopedic surgeon, a physical therapist, a health psychologist, and a group of research coordinators with experience recruiting patients with osteoarthritis. Experts attended a mock coaching session and provided input in a semistructured interview. Specific suggestions were extracted from the interview notes and used to refine the coaching intervention organization, content, and support materials. Experts’ suggestions primarily focused on the best way to communicate sensitive topics during coaching and the framing of patient predictions of likely outcomes if surgery was elected. For the former, clinicians encouraged the use of positive language, such as referring to comorbidities as “areas for possible improvement to optimize surgical outcomes.” For the latter, they suggested emphasizing that a good surgical outcome is one that improves arthritis pain and stiffness, but may not necessarily result in a perfect joint. Experts also offered advice on addressing the needs of nonsurgical patients including the importance of understanding personal data and setting goals regardless of one’s treatment plan.

Mock sessions using the refined intervention were then scheduled with ten patient advisers with hip or knee osteoarthritis recruited through the Arthritis Foundation. Patient advisers attended a session and provided feedback in either a one-on-one interview or written format. Generally, their feedback was positive. Most found reviewing the report during coaching to be helpful because they did not understand the PRO data on their own, and almost all mentioned valuing the supportive environment in which to discuss their concerns. Patient advisers were also enthusiastic about the “I Have a Voice” handout. Small changes to language and emphasis were made based on the patient advisor’s feedback, but the overarching session goals and content did not change.

The coaching intervention was launched for ASK study participants in early May 2020. Each initial session was staffed by two research team members: (1) the health educator who facilitated the session and (2) a research coordinator who gathered feedback from observations and provided technological support to attendees as needed. At the end of the first month of coaching, minor logistical changes were made, including adding answers to frequently asked questions and clarifying the instructions on joining a meeting via telecommunication platform Zoom (Zoom Video Communications). No additional changes were made after the first month. An outline of the final coaching session content is presented in Textbox 1.

**Textbox 1. The components of the final Arthritis care through Shared Knowledge (ASK) coaching session embedded within a pragmatic cluster-randomized trial for patients with hip and knee osteoarthritis.**

**Presession Materials (Mailed and Emailed to Participants):**

- Personalized ASK report (Multimedia Appendix 1)
- “I Have a Voice” handout (Multimedia Appendix 2)
- Instructions on how to join the meeting using the telecommunication platform (Zoom)
- Paper copy of the session slides

**Session Outline (1 h Online, Group):**

- Introductions (facilitator and attendees)
- Review of session goals
- Explanation of the importance of patient self-advocacy
- Detailed review of the ASK report and preparation to use the data in conversations with clinicians
  - Report section: Current pain and function scores
  - Reflection prompt: Is there anything that you might like to tell your clinicians about your pain and function levels?
  - Report section: Patient comorbidities
  - Reflection prompt: What aspects of your health and wellness would you like to address with your health care team, and what questions do you have?
  - Report section: Predictions of postsurgical pain and function
  - Reflection prompt: What are your goals for improvement post osteoarthritis treatment (including nonsurgical treatment)?
  - Report section: Nonsurgical treatment options
  - Reflection prompt: Are there any treatments about which you’d like to request more information?
- Additional strategies for a successful appointment
  - Planning to bring a trusted other
  - Asking to record clinicians’ instructions
- Review of additional resources

### Recruitment for the Coaching Intervention

**Figure 2.**
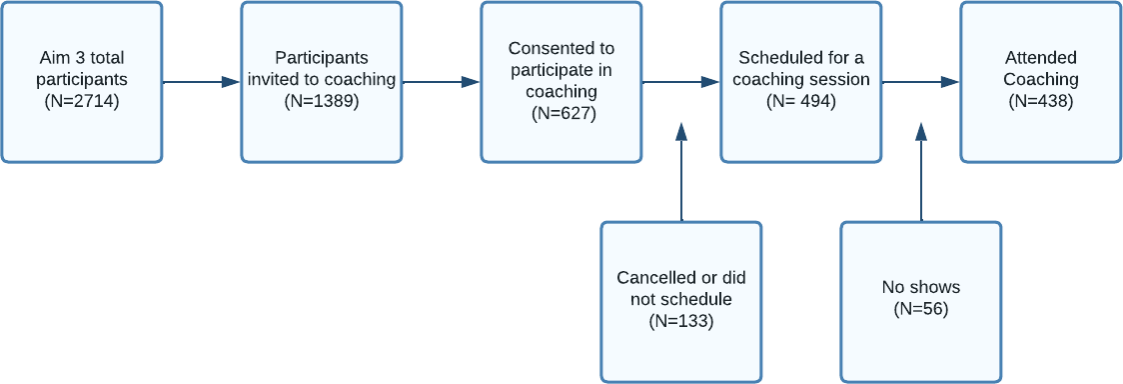
Attendance metrics for ASK coaching from May 2020 through February 2022 within a pragmatic cluster-randomized trial for patients with hip and knee osteoarthritis. ASK: arthritis care through shared knowledge.

All participants in the study’s coaching arm were invited to attend the online, group coaching session at the time of the postconsultation telephone survey. Multiple sessions were scheduled each week at varying times to accommodate patient schedules. Those who agreed to participate were scheduled for a session and both mailed and emailed the presession materials (Figure 2). While all study participants in this aim received a copy of their ASK report, only those who agreed to participate in the coaching session received the other materials. Participants scheduled for a coaching session received a reminder the day before, which included the number of a dedicated research coordinator who they could contact for technical support before or during the session.

Coaching consent and attendance rates were tracked throughout. Of all patients invited to coaching (n=1389), 45% consented to participate, and 32% of the total (N=438) attended a scheduled session. Among those who consented, 21% either did not schedule a session at the time of consent or scheduled a session but canceled, and 9% scheduled a session but did not show. Figure 2 summarizes attendance and cancellation rates. Research coordinators recorded reasons for declining to attend coaching, which included participants feeling they either did not need additional information about the report or their arthritis symptoms or were too busy to attend. Sessions had an average of 3‐4 attendees each, and enrollment extended from May 2020 through February 2022, which overlapped with the peak of the COVID-19 epidemic.

### Quantitative Evaluation of the Coaching Intervention

The timing of the quantitative coaching assessments within the broader aim 3 study flow can be found in Figure 1. To understand the profile of patients who attended coaching, sociodemographic information and clinical comorbidity data were obtained from all participants at sites in the study’s coaching arm and were descriptively summarized and compared by coaching attendance status using chi-square and 2-sample *t* tests that accounted for possible unequal variance. PROs included the Hip disability and Osteoarthritis Outcome Score (HOOS) or Knee injury and Osteoarthritis Outcome Score (KOOS-12) [24,25], osteoarthritis-specific PROs used broadly in research and clinical care, and the Veterans RAND 12-Item Health Survey (VR-12) [26] that assesses global physical and emotional function. Patients also self-reported pain intensity in other joints and lower back, sociodemographic factors, and medical comorbidities. A question to assess confidence in completing health forms screened for health literacy [27]. In late March 2021, roughly 1 year after the launch of ASK coaching, a patient-reported assessment of self-efficacy was added to the postconsultation telephone survey (ie, the time of the coaching invitation) to further understand the profile of those who did or did not choose to attend coaching sessions. This included 4 treatment-related items from the PROMIS (Patient-Reported Outcomes Measurement Information System) v1.0 Self-Efficacy for Managing Medications and Treatments [28] item bank (items SEMMT011, SEMMT012, SEMMT013, and SEMMT014), and was administered to the last 692 participants invited to coaching. Raw scores on this custom short form were converted to T-scores using the Health Measures Scoring Service. The T-scores were compared between those who attended and did not attend coaching using a Mann-Whitney *U* test as the scores were not normally distributed. A 2-tailed α level of <.05 was considered statistically significant for all inferential analyses.

In addition, to quantitatively assess attendee learning from coaching, a postsession survey was added in late March 2021. It included five self-assessment questions related to perceived change in knowledge and was sent via email after the coaching session. Responses to the self-assessment questions were descriptively summarized.

### Qualitative Evaluation of the Coaching Intervention

Data were integrated from multiple sources to qualitatively evaluate the ASK coaching intervention. First, questions related to acceptability and perceived benefits of coaching were included in semistructured interviews conducted in April-July 2021 as part of the parent study; 16 of these 25 interview participants had attended a coaching session. Only coaching-related segments from these 16 interviews were included in the present analysis; findings from other qualitative analyses of these interviews have been previously published [17,18]. Second, paired pre- and postcoaching interviews were conducted for 4 additional attendees in February 2022. These interviews provided finer-grained feedback on how coaching influenced participants’ understanding and potential use of PROs including in conversations with providers. All interviews were completed by a trained interviewer via Zoom and used a semistructured interview guide; they were audio-recorded and transcribed. Third, qualitative data were extracted from optional free-text responses provided by 47 coaching attendees in the postsession survey described above. Refer to Figure 1 for positioning of the qualitative assessments within the broader aim 3 study flow.

We used a qualitative descriptive approach to analysis, which aligned with our pragmatic and focused qualitative aims to learn more about what motivated participants to attend coaching sessions, assess the acceptability of an online group coaching session, and evaluate whether the session met the goals laid out by the research team [29-32]. After independent reading and preliminary coding of the 4 paired pre- and postcoaching interview transcripts, MB and BZS collaboratively developed a deductive codebook based on the qualitative aims. To increase trustworthiness, all interview transcripts and survey comments were dually coded by MB and BZS, with ongoing meetings to discuss emerging findings, iteratively refine the codebook, and resolve discrepant coding.

### Ethical Considerations

To ensure informed consent, all participants in the parent study were provided with a detailed consent form outlining the study’s procedures, potential risks and benefits, and their right to withdraw at any time. Participants in the coaching arm received an additional fact sheet with details about the coaching session, as did those who were invited to participate in an interview. Participants were paid US$ 20 for completing the postconsultation telephone survey as part of the parent study and an additional US$ 20 if they participated in an interview. No additional incentives were offered for participating in coaching or completing the postsession survey. All data collected as part of this study were deidentified and stored on a secure server. All study procedures were approved by the University of Massachusetts Medical School institutional review board (H00012297).

## Results

### Quantitative Evaluation of the Coaching Intervention

Sociodemographic and clinical profiles of coaching attendees versus nonattendees are summarized in Table 1. Compared with nonattendees, patients reporting higher education and greater health literacy were more likely to attend a coaching session when invited (*P*<.001). In addition, those with Medicare insurance were more likely to attend while those with Medicaid insurance were less likely to attend (*P*<.001). Medicare and Medicaid are US government–run health insurance programs. Medicaid provides insurance to low-income families and individuals. Medicare provides insurance to senior citizens and some individuals with disabilities. While there was a statistically significant between-group difference for age (*P*=.002), the mean difference was less than 2 years between those who attended versus those who did not. Furthermore, while there were no statistically significant differences, those who attended coaching were more likely to be female, have at least one comorbidity, and have slightly less preconsultation pain (Table 1). In addition, the subsample of participants with self-efficacy data, self-efficacy for managing treatments was lower in those who attended coaching (n=166; median T-score 48.6, IQR 41.8-57.3) than those who did not (n=526; median T-score 57.3, IQR 42.7-57.3; *P*=.05).

The postsession self-assessment of learning was completed by 89 coaching attendees (49% of those invited to respond). Approximately, two-thirds of respondents reported greater understanding of their current and projected symptoms. Along with this, three-quarters of participants reported more knowledge of where to find additional osteoarthritis information after coaching (Table 2).

**Table 1.** Sociodemographic and clinical profiles, stratified by coaching attendance status, of participants in the arthritis care through shared knowledge (ASK) coaching arm of a pragmatic cluster-randomized trial in patients with hip and knee osteoarthritis (May 2020-February 2022).

Patient characteristics	Total in coaching arm (n=1545)	Attended coaching (n=438)	Invited, but did not attend (n=1107)	*P* value^a^
Age (years), mean (SD)	66.5 (9.4)	67.7 (8.7)	66.1 (9.7)	.002
BMI, mean (SD)	31.0 (8.9)	30.7 (6.7)	31.1 (9.6)	.33
Preconsultation HOOS^b^ or KOOS-12^c^ Pain Score, mean (SD)	42.6 (16.3)	43.9 (15.2)	42.1 (16.7)	.05
Preconsultation HOOS or KOOS-12 ADL^d^ Score, mean (SD)	51.4 (20.4)	52.4 (19.3)	51.0 (20.7)	.20
Preconsultation VR-12^e^ Mental Component Summary Score, mean (SD)	55.8 (11.2)	56.4 (10.7)	55.6 (11.4)	.18
Preconsultation VR-12 Physical Component Summary Score, mean (SD)	31.7 (10.1)	32.0 (9.8)	31.5 (10.2)	.42
Sex, n (%)				
Male	572 (37.2)	146 (33.5)	426 (38.7)	.06
Female	966 (62.8)	290 (66.4)	676 (61.3)	
Race and ethnicity, n (%)				
Non-Hispanic White	1265 (85.8)	370 (87.5)	895 (85.2)	.70
Non-Hispanic Black	127 (8.6)	33 (7.8)	94 (8.9)	
Non-Hispanic other	30 (2.0)	7 (1.7)	23 (2.2)	
Hispanic	52 (3.5)	13 (3.1)	39 (3.7)	
Education, n (%)				
College graduate or postgraduate	862 (56.3)	296 (68.4)	566 (51.6)	<.001
Trade, technical school, or some college	363 (23.7)	79 (18.2)	284 (25.9)	
High school or less	263 (17.2)	47 (10.9)	216 (19.7)	
Other	42 (2.8)	11 (2.5)	31 (2.8)	
Confidence filling out medical forms (Health literacy), n (%)				
Not at all confident	14 (0.9)	1 (0.2)	12 (1.2)	<.001
A little bit confident	22 (1.4)	2 (0.5)	20 (1.8)	
Somewhat confident	75 (4.9)	8 (1.8)	67 (6.1)	
Quite a bit confident	253 (16.4)	72 (16.4)	181 (16.4)	
Extremely confident	1178 (76.4)	355 (81.1)	323 (74.5)	
Live with another adult, n (%)				
No, I live alone	424 (27.9)	127 (29.3)	297 (27.3)	.44
Yes, I live with another adult	1098 (72.1)	307 (70.7)	791 (72.7)	
Current marital status, n (%)				
Married	920 (59.8)	260 (59.6)	660 (59.9)	.93
Not married	618 (40.2)	176 (40.4)	442 (40.1)	
Health insurance, n (%)				
Private	594 (38.4)	161 (36.8)	433 (39.1)	<.001
Medicaid^f^	138 (8.9)	22 (5.0)	116 (10.5)	
Medicare^f^	717 (46.4)	238 (54.3)	479 (43.3)	
Other, Missing, None	96 (6.2)	17 (3.9)	79 (7.1)	
Sum of comorbidities, n (%)				
No comorbidities	749 (48.6)	193 (44.3)	556 (50.3)	.11
1 comorbid diagnosis	446 (28.9)	144 (33.0)	302 (27.3)	
2‐5 comorbid diagnoses	337 (21.9)	97 (22.3)	240 (21.7)	
6‐20 comorbid diagnoses	9 (.06)	2 (0.5)	7 (0.6)	
Low back pain, n (%)				
No or very mild back pain	1,076 (69.7)	304 (69.6)	772 (69.7)	.95
Moderate to worst imaginable pain	468 (30.3)	133 (30.4)	335 (30.3)	
Number of other hip or knee joints with moderate-severe pain, n (%)				
0	774 (50.1)	222 (50.7)	552 (49.9)	.76
1	560 (36.3)	151 (34.5)	409 (37.0)	
2	142 (9.2)	44 (10.1)	98 (8.9)	
3	69 (4.5)	21 (4.8)	48 (4.3)	

a Comparison using *χ*2 test or two-sample T-test between attendees and nonattendees.

b HOOS: Hip disability and Osteoarthritis Outcome Score.

c KOOS-12: Knee injury and Osteoarthritis Outcome Score.

d ADL: activities of daily living.

e VR-12: Veterans RAND 12-Item Health Survey.

f Medicare and Medicaid are US government-run health insurance programs. Medicaid provides insurance to low-income families and individuals. Medicare provides insurance to senior citizens and some individuals with disabilities.

**Table 2.** Postsession self-assessment of learning from a subsample of arthritis care through shared knowledge coaching attendees in a pragmatic cluster-randomized of patients with hip and knee osteoarthritis (March 2021-February 2022; N=89).

Compared with before attending coaching	Response (responses: n, %)
... I know how my pain and function compare to others like me.^a^	I know less now (n=1, 1.14%) I know about the same (n=20, 22.73%) I know more now (n=67, 76.13%)
... I know how my pain and function might change if I choose surgery.^a^	I know less now (n=1, 1.14%) I know about the same (n=18, 20.45%) I know more now (n=69, 78.11%)
... I know what outcomes I can expect if I choose surgery.^a^	I know less now (n=0, 0%) I know about the same (n=23, 26.13%) I know more now (n=65, 73.87%)
... I know what other aspects of my health impact my arthritis.	I know less now (n=1, 1.12%) I know about the same (n=25, 28.09%) I know more now (n=63, 70.99%)
... I know where to find information about caring for my arthritis.	I know less now (n=1, 1.12%) I know about the same (n=9, 10.11%) I know more now (n=79, 88.76%)

a Missing for one respondent.

### Qualitative Evaluation of the Coaching Intervention

The qualitative evaluation results are organized into participants’ perspectives on motivation for attendance, acceptability of session format, achievement of session goals, and suggestions for improvement. Additional supporting quotes for each subsection are presented in Multimedia Appendix 3.

#### Motivation for Attendance

Participants’ main motivations for attending ASK coaching were to gain information that could benefit their treatment and to aid in research that could help others. Specifically, attendees expressed interest in improving their understanding of the ASK report (including PROs), getting more information about their condition, developing strategies for future conversations with clinicians, and learning from peers.

*I think because I was so confused by that first page [of PROs in the report], I wanted some more explanation as to if I was understanding it correctly or how to understand it*.

[P09, 70 y.o. White, Female]

#### Acceptability of the Session Format

The online format of the session was generally acceptable to participants although some would have preferred to meet in person had that option been available. Several participants expressed challenges with the online platform and valued the technical support offered by the ASK team.

*I thought [the session] was very good. I mean even having an IT person on just in case anybody had trouble with Zoom. I mean it feels like you’ve thought of everything*.

[P04, 59 y.o. White, Female]

The group nature of ASK coaching was also generally acceptable. It was even noted as specifically valuable by participants who valued hearing experiences of others, such as those farther along in the treatment process. This finding aligned well with early feedback from the design process positing that a group format would allow for the sharing of experiences. However, there were some divergent opinions, with two participants who had more advanced osteoarthritis noting that differing disease severity and treatment plans among their fellow participants negatively impacted their session experience. One participant highlighted that the small group format was acceptable at her current age but may not have been when she was younger because of social discomfort discussing health. Several participants also pointed out that certain group members negatively impacted group dynamics but did not feel that was a reason to discontinue group offerings.

*I liked the group format of it, and maybe it’s because I am on the earlier end of this condition, and the other participants were all giving me advice. ... You get that little sense of camaraderie. Everybody’s rooting for one another*.

[P06, 57 y.o. White, Female]

The presence of a facilitator was discussed by many as important to the acceptability of the format, since it provided structure and focus to the session. Participants highlighted specific benefits of having a health educator as the facilitator, including the opportunity to ask health-related questions from someone outside of their immediate health care team.

*I appreciate the personal, stress-free atmosphere in which I felt totally comfortable asking questions I would never ask my doctor in the interest of time or seeming uninformed*.

[P11, 59 y.o. Black, Female]

#### Achievement of Session Goals

In line with the session goal of helping participants to understand their personal data, coaching attendees reported gaining a better understanding of the ASK report, including their PRO data. Several discussed that coaching participation improved report understanding and clarified misinterpretations of their data even if they had not experienced initial confusion.

*I’m just arrogant enough to believe that I understood [the report] thoroughly because I read it carefully. ...And I found at the end of the session I was really glad that I had participated. And maybe it’s a little bit like when you teach a person to write, you say, ‘First tell them what you’re gonna say. Then say it. Then tell them what you’ve said.’ So, I think having read it, and then having gone through it more carefully, hearing someone else say it, I was surprised at just how useful [the session] was. I did think I understood [the report] much better.*

[P07, 77 y.o. White, Female]

Related to the goal of preparing for future conversations with clinicians, patients reported that participating in ASK coaching helped them feel more confident speaking with clinicians and asking questions. The “I Have a Voice” handout, which was intended to support this, was well-liked and even named as the best part of the session by some participants. Participants noted that the additional resources provided also helped them feel more prepared for such conversations.

*‘I Have a Voice.’ Absolutely fabulous. ... And in hearing [the health educator’s] presentation, it gave us things to think about to ask. So, it was the most helpful part I think of the talk was that ‘I Have a Voice.’ Because it’s true. ... And [the study surgeon] is great. He asked me some of these questions. But I just, it gave me ideas of things to ask after, when I see him again.*

[P09, 70 y.o. White, Female]

*I do plan on going to the ASK website and to the Arthritis Foundation website either this evening or tomorrow and drilling down a little bit on treatments. ... So, I just want to get more information, so in having that resource, puts me on a more equal footing with my doctor because if before my next appointment if I have more information, I can ask more intelligent questions when I see him and that could improve my treatment. So, it’s been good in that way.*

[P12, 73 y.o. White, Male]

#### Suggestions for Improvement

While participants overall described the acceptability and benefits of ASK coaching, they also offered suggestions for improvement. The most frequent request was to provide additional or more structured opportunities for patients to connect with each other inside and outside of the coaching session. Another suggestion was to create coaching groups based on patients’ previous experience with joint replacement surgery or stage of disease progression. One participant suggested spreading the material across 2 sessions for better understanding. In addition, given the pragmatic challenges of scheduling the coaching session before the surgeon consultation, another participant suggested having a recorded video to accompany the “I Have a Voice” handout allowing individuals to view it in preparation for the consultation.

## Discussion

### Overview

This paper describes the iterative design and evaluation of an online, group coaching session offered as a companion to a PRO-based decision aid for patients with hip or knee osteoarthritis who had an initial consultation with an orthopedic surgeon. The collaboratively designed session, referred to as ASK coaching, aimed to support patients in understanding their personal health data, including PRO scores, and using the data in future conversations with clinicians. Evaluation findings highlight differences in attendance based on sociodemographic characteristics and provide insight into motivation for attendance, session format acceptability, and achievement of session goals. Implications for potential design refinement, including priorities for future research, are integrated throughout the discussion.

### Principal Findings

Osteoarthritis of the knees and hips is one of the most common causes of chronic pain and disability in the United States. It currently affects more than 21 million Americans, with increasing prevalence as the population ages [33,34]. Osteoarthritis is a chronic condition for which there is no cure, and treatment options vary based on symptom severity and patient preference [35]. Therefore, patients’ understanding of their symptoms and ability to communicate with providers are particularly important. In today’s clinical practice, patients are accustomed to completing previsit assessments of their demographic, insurance, and health status information. However, receipt of their personal data is new to most patients, as is participating in a group coaching session to support the understanding and use of such data.

While patients routinely completed the previsit PROs, not all of them elected to participate in ASK Coaching, so this paper attempts to understand who this intervention may reach. First, patients reporting higher education and health literacy levels were more likely to join a session. This finding aligns with research showing that patients with more formal education and higher health literacy are more likely to engage in educational programming related to their arthritis care [36], a pattern that can contribute to the exacerbation of inequities if those with the greatest need for support are the least likely to access it. In contrast to high literacy and education, ASK coaching attendees reported lower confidence in managing their treatment as compared to nonattendees, suggesting they may have appropriately self-selected to attend based on their perceived need. Coaching attendees were also less likely to have Medicaid insurance than nonattendees, which may reflect a lack of access to health education or digital interventions among those with lower socioeconomic status [37]. In addition, coaching attendees were slightly older than nonattendees. While this difference in age was likely not clinically meaningful, it provides reassurance that older adults can readily and successfully attend an online session [38]. While there was no significant between-group difference for sex, patients reporting female sex tended to more often attend ASK coaching, which is consistent with existing literature showing women are more likely to seek out health information than men [39,40]. Patients with at least one comorbidity were more likely to attend a coaching session when invited, which may indicate those managing multiple competing health priorities have a greater need for support in preparing for conversations with clinicians. Interestingly, those with slightly lower preconsultation pain were also more likely to attend, which may indicate pain can be a barrier to engaging with supportive resources [41]. Priorities for future research are identifying for whom this intervention is most beneficial, more deeply understanding barriers and facilitators to attendance in subgroups at risk of intervention-generated inequality [37], and refining the intervention design to address those subgroup needs.

A primary motivation for coaching attendance was to gain additional information. Relatedly, the most common reason for not attending was having adequate information. While not all patients may require a resource like ASK coaching to understand PRO data and engage in conversations about their osteoarthritis care, patients also may not initially recognize gaps in their own knowledge. Qualitative findings reinforced patient-perceived benefits regarding gaining information about their PRO data even when they initially reported understanding. Similarly, previous qualitative research about the ASK report highlighted misinterpretations that were not always recognized by participants [18]. Future research could therefore explore how to best communicate the potential value of participation, even to those who feel confident in their knowledge, through co-design of presession materials. A second reason study participants did not join ASK coaching was that they reported feeling too busy. Time is a common barrier when it comes to engaging participants in health promotion efforts [42,43]. While this research was conducted during the COVID-19 pandemic, a particularly challenging time for all, limited patient time supports further refinement of the session length and the development of an asynchronous option to reduce barriers to attendance.

This paper also explores the acceptability and potential effectiveness of coaching patients to use a PRO-based decision report. Among attendees, the online format, with technological support provided as needed, was generally accepted. While some preferred in-person meetings, if possible, online sessions like this one may be easier to implement as they have been shown to be more cost-effective [44], particularly for health systems or case management programs that serve a large geographic area. Most attendees also endorsed the small group nature of ASK coaching. However, qualitative feedback made it clear that this structure could be improved to maximize the session’s benefits by including more unstructured time to ask questions and interact with peers. Given constraints related to clinician availability in many health systems, especially for nonreimbursable educational activities, it is worth noting participants found the nonclinician health educator to be an acceptable professional facilitator. However, adding a peer educator could benefit those interested in peer support, which has been shown to have benefits for other chronic conditions [45,46]. Based on these findings, a variety of design modifications could be evaluated in future research. To encourage participation among those who prefer in-person programs, the intervention design could be refined to improve the experience, including more scalable technological support outside of a research coordinator. Based on feedback about disparate participant experience, groups could also be organized by current treatment plans, such as surgery or nonsurgical treatments, to ensure the discussion is relevant for all. It should be noted, however, that defining groups in this way would limit attendees’ ability to learn from people who were further along in the treatment process, something coaching participants appreciated. Additional options for future intervention refinement include splitting the session into multiple shorter sessions or providing information that could be viewed asynchronously before a moderated discussion, both of which would offer greater opportunities for interaction while addressing time constraints.

With regards to session goals, attendees reported that ASK coaching provided clarification of specific PRO graphs and improved overall report comprehension. Previous publications of patients’ perspectives of the ASK report highlighted challenges with report comprehension, which support the role of coaching [17,18]. The session goal of preparing patients for future conversations with clinicians was also primarily met, with many patients expressing that they felt more ready to speak up and ask questions, finding the “I Have a Voice” handout particularly helpful. These positive findings related to acceptability and preliminary effectiveness were likely a function of the iterative design process, highlighting the value of collaboration with both clinical experts and patients during design to align the intervention to the users’ needs.

Additional implications for design and future research include considerations related to both efficacy and scalability. The timing of the session within the parent study limited patients’ ability to immediately use the information and skills from the session. When the study was designed, the return of PRO data directly to patients was not the norm in clinical care. Thus, the parent study proposed that the ASK report would be distributed in the surgeon’s office enabling each clinician to review the information with their patients. The online coaching session then followed this surgeon consultation and was intended to provide a second, more detailed review of the report. While the design worked well within the established care structure, it meant that participants had a clinical consultation before attending the session. Given that PROs are more commonly included in clinical care today, and participants felt the session helped increase their confidence when communicating with clinicians, it is likely that a greater benefit would have been achieved had the session taken place prior to the initial surgeon consultation. At a minimum, participants reported that receiving information about the “I Have a Voice” handout in advance of that initial appointment would have been helpful, aligning with the principles of decision guidance—supportive elements embedded within or alongside the decision aid for independent use [22]. In addition, providing this patient preparation resource could benefit clinicians who only have a short time for each appointment by ensuring that patients can quickly share details about their arthritis and its impact on their lives [47]. Combining this handout with a prerecorded asynchronous version of the coaching session would likely also improve this intervention’s scalability as it is a one-time cost and does not require additional clinician time or onsite resources. However, this intervention format may not address all patient needs, such as being able to ask questions of a trained facilitator and interacting with other arthritis patients. In future efforts, the research team will propose a real-time return of the ASK report and coaching before the initial surgeon consultation to support patient preparation for that initial treatment discussion. Additional research in collaboration with clinicians and patient experts will further refine the design of coaching and its implementation, including asynchronous or hybrid versions that align with both patient and health system needs.

### Limitations

While the parent study enrolled both English-speaking and Spanish-speaking participants, the coaching session was only offered in English, potentially limiting the generalizability of findings. Also, many participants who chose to attend the session were well-educated about arthritis and treatment options and therefore may have been less likely to benefit from participating. This limits conclusions about the intervention’s effectiveness. While there were no significant racial or ethnic differences in coaching attendance, study participants were largely White, reflecting well-documented racial and ethnic disparities in referrals to orthopedists for joint replacement surgery [10]. Coaching acceptability may differ for racial or ethnic minorities and needs to be examined in future research with more diverse samples, given the potential benefits of participating in an intervention of this nature to reduce inequities in osteoarthritis care. Furthermore, patients with a strong preference for a different session format may have simply chosen to not attend, and beyond asking participants who declined to participate why they were not interested, the research team did not seek more detailed feedback. As such, it is difficult to draw generalizable conclusions about session format acceptability. ASK coaching was also part of a complex health intervention that included the PRO-based report and consultation with the surgeon. This complexity makes it challenging to parse out specific benefits of coaching. Moreover, different components of the quantitative and qualitative evaluation were pragmatically added at various points in this multi-method evaluation, limiting the ability to systematically integrate such data using a mixed methods analysis. Advanced data integration may have been helpful to provide additional insight into which patient subgroups might benefit the most from this intervention. While we designed the intervention for efficiency (eg, group visits and online coach), we did not formally evaluate the intervention’s costs, which could be a barrier to broad implementation in routine care. Finally, this portion of the study took place during the COVID-19 pandemic, which may have influenced patients’ perspectives on the online and group components of the coaching format.

### Conclusions

This paper describes the iterative design and evaluation of an online coaching intervention delivered by a health educator to support patients in understanding and using a novel PRO-based report as part of hip or knee osteoarthritis care, including in conversations with clinicians. Among coaching attendees, the acceptability of the session format was overall high, and session goals were achieved. With some modifications related to timing and structure, this intervention appears promising to support patients in using personal health data, including PRO scores, to prepare for conversations with their clinicians. Future research is needed to further refine this intervention and scale implementation, including ensuring reach to diverse patients.

## Supplementary material

10.2196/65931 Multimedia Appendix 1 Copy of the arthritis care through shared knowledge report.

10.2196/65931 Multimedia Appendix 2 “I Have a Voice” handout.

10.2196/65931 Multimedia Appendix 3 Additional supporting quotes for the qualitative evaluation results from interviews and free-text survey responses from a subsample of arthritis care through shared knowledge (ASK) coaching attendees in the pragmatic cluster-randomized ASK trial in patients with hip and knee osteoarthritis.

## Abbreviations

AHRQ: Agency for Healthcare Research and Quality
ASK: arthritis care through shared knowledge
CPS: Citizens for Patient Safety
HOOS: Hip disability and Osteoarthritis Outcome Score
KOOS-12: Knee injury and Osteoarthritis Outcome Score
PRO: Patient Report Outcome
PROMIS: Patient-Reported Outcomes Measurement Information System
VR-12: Veterans RAND 12-Item Health Survey
