# Supplementary material for: Coaching Patients to Understand and Use Patient-Reported Outcome Data: Intervention Design and Evaluation
Source: JMIR Form Res. 2025 Jun 30;9:e65931. doi: 10.2196/65931 (PMC12261967; doi:10.2196/65931)
Supplement: Multimedia Appendix 2 [file formative-v9-e65931-s002.pdf]

I Have A Voice

Your doctors, nurses, physical therapists, and surgeons all want you to know that you are an important member of the healthcare team. What you are thinking and feeling matters. You have a voice and they want you to use it!

The following prompts can help you prepare to be an active participant at your next appointment. You have information to share and questions to ask, and writing those down in advance can help you remember what you need to say.

I have information

Your healthcare provider knows that you're the expert on you, and wants you to share information about how are you feeling, and the changes you'd like to see. What would you like your healthcare provider to know?

1. My arthritis is causing the following challenges:

____

____

2. My goals for my health are:

____

____

3. After treatment, I want to be able to:

____ _

____

4. This other factor may also be impacting my health outcomes:

____

____

I have choices

5. There are many ways to treat arthritis pain. Some may be more interesting to you than others. Your healthcare provider has information about which treatment will work best for you. Which options would you like to talk to them about?

____

____

____

____

I have questions

6. When it comes to communicating with your healthcare provide, questions are the answer! You can start a list of what you want to ask, and who you will plan on asking, below:

Question: ____

____
____

Healthcare provider I will ask: ____

Question: ____

____
____

Healthcare provider I will ask: ____

Question: ____

____
____

Healthcare provider I will ask: ____

Question: ____

____
____

Healthcare provider I will ask: ____

7. Asking questions is great, but it can be challenging to remember the answers. You can write notes here during your appointment so that you don't forget what your healthcare provider has told you:

____
____
____
____
____
____
____
____

I have support

Medical appointments can be stressful, even with preparation. Many people find that bringing a supportive friend or relative helps them be more comfortable using their voice. Is there anyone you would like to bring to your appointment?

8. I would like to bring:

____
