# Supplementary material for: Coaching Patients to Understand and Use Patient-Reported Outcome Data: Intervention Design and Evaluation
Source: JMIR Form Res. 2025 Jun 30;9:e65931. doi: 10.2196/65931 (PMC12261967; doi:10.2196/65931)
Supplement: Multimedia Appendix 3 [file formative-v9-e65931-s003.docx]

Additional supporting quotes for the qualitative evaluation results from interviews and free-text survey responses from a subsample of A.S.K. Coaching attendees in the pragmatic cluster-randomized A.S.K trial in patients with hip and knee osteoarthritis

| **Motivation for Attendance** | |
| --- | --- |
|  | “I wanted to get more insight into the disease itself and how to manage it and as I go through the decision making if I’m gonna have the surgery or not.” (P12, 73 y.o. White, Male) |
|  | “I think I really wanted to hear from other people. And it was most helpful to hear from another person that is going through some of the same stuff that I’m going through. So, yeah, it’s a misery loves company kind of thing.” (P14, 71 y.o. White, Female) |
| **Acceptability of the Session Format** | |
| Virtual |  |
|  | “I always prefer face-to-face, but real face-to-face. Zoom is a close second. It’s gotten better over the years and circumstances in the last 2.5 years have made Zoom almost a default for face-to-face meetings, but I just prefer the human interaction and being out of the house basically.” (P12, 73 y.o. White, Male) |
| Group |  |
|  | “And it’s interesting to me the perspectives that other people bring. A lot of times other people think of things that I certainly haven’t thought about. Sometimes I would rather not have thought of some of the things other people think about. But it’s kind of like better to have it out on the table and not come up with something in the middle of the night and not be able to talk about it with other people.” (P08, 65 y.o. White, Female) |
| Facilitator |  |
|  | “I just think that the fact that someone was a dedicated person to come in and actually facilitate this discussion of my health with me and another person. That was really the most valuable thing. I think we don’t want to talk a lot about their health problems because then nobody wants to talk to you because you’re just overwhelming. So, it was good to have a place where I could focus on that and not have to say, ‘I’m only giving myself 10 minutes to talk about this and that’s it.’ So, I really think that was very, very helpful.” (P14, 71 y.o. White, Female) |
| **Achievement of Session Goals** | |
| Report Understanding |  |
|  | “When I first looked at it [the report], I said, ‘What’s this all mean?’ So, when we stopped and kind of broke it down, I think it was helpful for me to understand what the meaning of these different charts and graphs were.” (P03, 72 y.o. White, Male) |
|  | “I think it did have an effect because I was really saying, ‘I’m not gonna get a knee replacement. I’m not getting it replaced. I’m gonna do everything I can not to do that.’ And after talking through it more and having the graphs explained again, it sort of reinforced that I’d probably would have a pretty good outcome if I were to have that done. And so, I probably won’t put it off.” (P14, 71 y.o. White, Female) |
| Preparing for Conversations with Providers |  |
|  | “I think that [the session] gave me food for thought that I can actually think more and come up with some questions, mainly, of course, about the outcome, my outcomes, and then also about the pain medication, pain management. Also, probably a little bit in regards to the home recovery part of it. ... It just helped me to give me more confidence to be able to talk to [my surgeon] about what might need to come down the road and what types of things can go on. He seemed like he was able to talk with me however long I wanted to go and talk. The problem was that I tend to get white-coat – even though I’m a nurse, I still get white-coat anxiety kind of thing. And it just kind of blows me away for a few minutes, and I can’t think of anything. So, it just takes me a while. So, this has given me some time to think about what I would ask.” (P14, 71 y.o. White, Female) |
|  | “I think after the coaching session I feel more comfortable and knowledgeable about questions that I can ask at my doctor’s appointment.” (P10, 68 y.o. White, Female) |
| **Suggestions for Improvement** | |
|  | “I just would really, really appreciate the opportunity to work with other people that have done this already, and maybe people that are working on doing this. Like I’d like to meet some of the people that are of that 3000 or 6000 people that were on that graph ... I’d like to hear some of their stories, and I’d like to do more group work, and exchanging phone numbers, if that’s something that people would be interested in.” (P13, 43 y.o. White, Male) |
|  | “I think it's good to have it in two days. There's so much information. It's like it's a lot to know from— without a medical background, there's a lot to figure out.” (P15, 70 y.o. White, Female) |
|  | “In some ways, people didn't really have a chance to talk. But in other ways it would have been too long had they. I think it was an hour, and that was enough time. But I don't think it was enough time for each person. Maybe they have two levels, levels of people who this is their first time ... with surgery .... And then, another group of more seasoned people as it were.” (P05, 73 y.o. White, Female) |
|  | “I think that it would be helpful if you had a link that you could send out to people to watch about the 'I Have a Voice' paper. ... Because when you go in, you don’t know what you don’t know. I mean, I’ve never had replacement surgery. So, I don’t know what questions to ask. So, it’s helpful.” (P09, 70 y.o. White, Female). |
| A.S.K = Arthritis care through Shared Knowledge | |
